# Retraction Note: Pericyte loss influences Alzheimer-like neurodegeneration in mice

**DOI:** 10.1038/s41467-024-47285-6

**Published:** 2024-04-03

**Authors:** Abhay P. Sagare, Robert D. Bell, Zhen Zhao, Qingyi Ma, Ethan A. Winkler, Anita Ramanathan, Berislav V. Zlokovic

**Affiliations:** 1 Department of Physiology and Biophysics, Keck School of Medicine, Zilkha Neurogenetic Institute, University of Southern California, Los Angeles, California 90033 USA; 2 Center of Neurodegenerative and Vascular Brain Disorders, University of Rochester Medical Center, Rochester, New York 14642 USA

Retraction to: *Nature Communications* 10.1038/ncomms3932, published online 13 December 2013

The authors have retracted this article after concerns were raised about some of the data reported. Similarities were identified between representative image panels in Fig. 7, which shows tissue staining with four different anti-tau antibodies:

- Fig. 7e and 7f (hippocampus in control AppSW/0 Pdgfrb+/+ mice) images show similarities in negative staining for tau on tissue sections with two different tau antibodies.
- Fig. 7e and 7f (hippocampus in pericyte-deficient AppSW/0 Pdgfrb+/− mice) images show similarities in positive staining for tau on tissue sections with two different tau antibodies.
- Fig. 7a and 7e (hippocampus in control AppSW/0 Pdgfrb+/+ mice) images show similarities in negative staining for tau on tissue sections with two different tau antibodies.

Due to the age of the paper, the authors no longer have access to all original data for this publication, and so can no longer be confident in the reliability of the data reported in this article. All authors agree to this retraction.

